# Inflammation functions as a key mediator in the link between ACPA and erosion development: an association study in Clinically Suspect Arthralgia

**DOI:** 10.1186/s13075-018-1574-3

**Published:** 2018-05-03

**Authors:** R. M. ten Brinck, R. E. M. Toes, A. H. M. van der Helm–van Mil

**Affiliations:** 1 Department of Rheumatology, Leiden University Medical Center, PO Box 9600, Leiden, 2300RC the Netherlands; 2 Department of Rheumatology, Erasmus Medical Center, Rotterdam, The Netherlands

**Keywords:** Rheumatoid arthritis, Autoantibodies, Imaging, Inflammation

## Abstract

**Background:**

Anti-citrullinated protein antibodies (ACPA) are associated with more severe joint erosions in rheumatoid arthritis (RA), but the underlying mechanism is unclear. Recent in vitro and murine studies indicate that ACPAs can directly activate osteoclasts leading to bone erosions and pain. This study sought evidence for this hypothesis in humans and evaluated whether in patients with arthralgia who are at risk of RA, ACPA is associated with erosions (detected by magnetic resonance imaging (MRI)) independent of inflammation, and also independent of the presence of rheumatoid factor (RF).

**Methods:**

Patients with Clinically Suspect Arthralgia (n = 507) underwent determination of ACPA and RF and 1.5 T contrast-enhanced MRI of the metacarpophalangeal, wrist and metatarsophalangeal joints at baseline. MRIs were scored for presence of local inflammation and erosions. Comparisons of erosion scores were performed using the Kruskal-Wallis test. To evaluate if inflammation is, in statistical terms, intermediary in the causal path of ACPA and erosions, three-step mediation analysis was performed using linear regression.

**Results:**

ACPA-positive patients had higher erosion scores than ACPA-negative patients (*p* = 0.006). ACPA-positive patients without subclinical inflammation did not have higher erosion scores than ACPA-negative patients (*p* = 0.68), in contrast to ACPA-positive patients with local inflammation (*p* < 0.001). Mediation analyses suggested that local inflammation is in the causal path of ACPA leading to higher erosion scores. Compared to ACPA-negative/RF-negative patients, ACPA-positive/RF-negative patients did not differ (*p* = 0.30), but ACPA-positive/RF-positive patients had higher erosion scores (*p* = 0.006).

**Conclusions:**

The effect of ACPA on erosions is mediated by inflammation and is not independent of RF.

**Electronic supplementary material:**

The online version of this article (10.1186/s13075-018-1574-3) contains supplementary material, which is available to authorized users.

## Background

Although anti-citrullinated protein antibodies (ACPA) are the most important risk factor for joint destruction in rheumatoid arthritis (RA), the underlying pathophysiological process is unclear. Traditionally, it is hypothesized that ACPAs can enhance inflammation [1] (for instance via immune complexes that stimulate macrophages to secrete pro-inflammatory cytokines) and that inflammation is required for destruction, resulting in e.g. visible bone erosions on radiographs. Recent in vitro studies and mouse models have generated a new concept in which ACPA can directly induce osteoclast activation, followed by autocrine enhancement of osteoclast maturation and activation [2, 3]. This may subsequently lead to bone loss (and pain) as observed in studies performed in vivo following injection of ACPA [2–5]. The finding that ACPA can be present long before synovitis is clinically detectable [6] and that sensitive imaging techniques have detected small erosions in patients with arthralgia [7] fit with the hypothesis that joint inflammation is not necessary to generate erosions [5]. Despite observations made in vitro and in vivo in mice [2, 3], there is presently little information available on ACPA-positive patients in the absence of local inflammation. Hence, it is not known if ACPA can lead to bone erosions only with concurrent presence of inflammation, or that ACPA induces direct osteoclast activation (leading to erosions without requiring concomitant inflammation) in humans as well. By performing association studies in patients that are in the disease phase of arthralgia without the presence of clinical synovitis, information on these relationships can be obtained as only a proportion of the patients with arthralgia display subclinical inflammation. Therefore, the arthralgia setting provides the possibility to study associations between ACPA, (local) inflammation and erosions.

Likewise, this setting can also be used to answer whether the effect of ACPA - if any - is dependent on the presence of rheumatoid factor (RF). Studies within early (rheumatoid) arthritis, using high-resolution computed tomography (CT), have shown that combined presence of ACPA and RF is associated with the number and size of erosions rather than ACPA alone [8]. In addition, it has been shown that patients with early arthritis harboring both ACPA and RF display increased osteitis scores as detected by magnetic resonance imaging (MRI), in contrast to ACPA single-positive patients [9].

With the aim to find supporting evidence that ACPAs themselves are directly linked to bone erosions in humans, this study in patients with Clinically Suspect Arthralgia evaluated whether (1) ACPA were associated with higher erosion scores (detected by MRI) independent of the presence of inflammation, and (2) whether higher erosion scores were associated with ACPA alone or with ACPA and RF combined.

## Methods

### Patients

Patients with arthralgia (n = 507) consecutively included in the Leiden Clinically Suspect Arthralgia cohort between April 2012 and September 2017 were studied. Clinically Suspect Arthralgia (CSA) was defined as: recent-onset (< 1-year) arthralgia in small joints, without clinically detectable synovitis (i.e. joint swelling) on physical examination, while the treating rheumatologists considered the patients suspicious of progression to RA based on their clinical presentation [10]. General practitioners in our region rarely performed ACPA or RF testing before referral [11]; hence this infrequently affected inclusion decisions [10]. After inclusion, patients were considered ACPA positive (EliA cyclic citrullinated peptide (anti-CCP2), Phadia, Nieuwegein, the Netherlands) if levels ≥7 U/mL) were detected, RF positive (as described previously, in-house ELISA [12]) if levels ≥3.5 IU/mL were detected and C-reactive protein (CRP) positive if levels ≥5.0 mg/L were detected. The cohort has previously been described in detail [10]. Informed consent was obtained from all subjects. The local medical ethical committee approved the study.

Within 1–2 weeks after inclusion, patients underwent 1.5 T contrast-enhanced MRI of the 2nd to 5th metacarpophalangeal joints (MCP2–5), wrist and the 1st to 5th metatarsophalangeal joints (MTP1–5) of the most painful side (see Additional file 1: Methods for MRI protocol). Disease-modifying anti-rheumatic drugs were not used. Non-steroidal anti-inflammatory drugs (NSAIDs) were stopped 24 h before the patients underwent MRI. The MR images were scored for erosions, bone marrow edema (BME), synovitis [13] and tenosynovitis [14], by two readers as described in Additional file 1: Methods. Within-reader intraclass correlation coefficients (ICC) were 0.98 and 0.99; the between-reader ICC was 0.96.

### Inflammation

Inflammation was assessed in two ways: first, local inflammation was considered present if there was MRI-detected BME, synovitis or tenosynovitis that was more than that observed in age-matched symptom-free controls [15] in ≥ 1 joint (Additional file 1: Methods). Second “any inflammation” was defined as the presence of either local subclinical inflammation (MRI-detected synovitis, BME or tenosynovitis) and/or elevated CRP. In this second analysis, “any inflammation” (i.e. systemic inflammation) was taken into consideration as it could be argued that the presence of increased acute phase reactants in patients that have no subclinical joint inflammation as detected with MRI indicates that some inflammation is present in these patients.

### Analyses

Erosion scores were compared using the Kruskal-Wallis test. To evaluate if inflammation is, in statistical terms, intermediary in the causal path of ACPA and erosions, mediation analyses were performed as described by Baron and Kenny [16]. Here, linear regression was used to evaluate in three steps if local inflammation is a mediator in the causal path of ACPA presence and erosion score as outcome. First, the association between presence of ACPA and erosions was investigated. Second, the association between presence of ACPA and severity of local inflammation was investigated. Finally, both ACPA and local inflammation were entered into the model and we tested whether this effect was different from the association between ACPA alone and erosion score. The percentage of mediation was calculated. All regression analyses were corrected for age. Additionally, triple stratification was applied for ACPA, RF and local subclinical joint inflammation. The Statistical Package for the Social Sciences (SPSS) version 23.0 was used.

## Results

Patients with CSA had a mean age of 44 years, 77% were female and presence of local subclinical joint inflammation on MRI was observed in 50% of patients (*n* = 255): 64% of the patients included met the European League Against Rheumatism (EULAR) definition of arthralgia suspicious of progression to rheumatoid arthritis (3/7 items present) [17]. Further characteristics are shown in Table 1.

**Table 1:** Baseline characteristics of the patients with Clinically Suspect Arthralgia (*N* = 507)

Patient characteristic		
Age in years, mean (SD)	44	(13)
Female sex, *n* (%)	390	(77)
Family history of RA, *n* (%)	147	(29)
Symptom duration in weeks, median (IQR)	17	(9–32)
Presence of morning stiffness ≥60 min, *n* (%)	182	(36)
Current smoker, *n* (%)	137	(27)
68-TJC, median (IQR)	6	(3–10)
Increased CRP (≥5 mg/L), *n* (%)	106	(21)
Presence of local subclinical joint inflammation, *n* (%)	255	(50)
Positive for EULAR definition for arthralgia suspicious for progression to RA [17], *n* (%)	325	(64)
Autoantibody status		
Negative for IgM-RF and ACPA, *n* (%)	385	(76)
IgM-RF-positive (≥3.5 IU/mL), ACPA-negative, *n* (%)	52	(10)
ACPA-positive (≥7 U/mL), IgM-RF-negative, *n* (%)	15	(3)
IgM-RF-positive and ACPA-positive, *n* (%)	55	(11)
ACPA-level (U/ml) in ACPA-positive patients, median (IQR)	162	(35–340)
ACPA-level (U/ml) in ACPA-positive patients without local joint inflammation, median (IQR)	129	(23–340)
ACPA-level (U/ml) in ACPA-positive patients with local joint inflammation, median (IQR)	191	(38–340)

Local subclinical joint inflammation was identified if the prevalence of magnetic resonance imaging (MRI)-detected bone marrow edema, synovitis or tenosynovitis was higher than that of age-matched symptom-free controls

*ACPA* anti-citrullinated peptide antibody, *CRP* C-reactive protein, *EULAR* European League Against Rheumatism, *IgM-RF* immunoglobulin M rheumatoid factor, *IQR* interquartile range, *RA* rheumatoid arthritis, *RF* rheumatoid factor, *SD* standard deviation, *TJC* tender joint count

### ACPA with concomitant inflammation, but not ACPA alone, associated with higher erosion scores

**Fig. 1:**
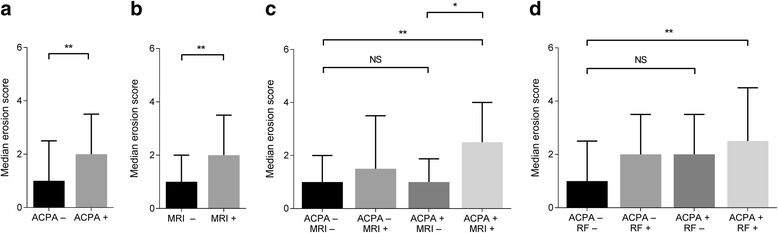
Histograms showing median erosion scores in patients with Clinically Suspect Arthralgia comparing anti-citrullinated protein antibodies (ACPA)-positive and ACPA-negative patients (**a**), patients positive or negative for local subclinical joint inflammation (**b**), ACPA positivity and negativity in relation to the concomitant presence of magnetic resonance imaging (MRI)-detected subclinical inflammation (**c**), or rheumatoid factor (**d**). Median erosion scores with the upper limit of the interquartile range (75th percentile): **p < 0.01; *p < 0.05; NS, non-significant. The following comparisons have been made: ACPA+ vs. ACPA− (**a**) (*p* = 0.006) and MRI+ vs. MRI− (**b**) (*p* < 0.001). Next, ACPA+MRI− vs. ACPA–MRI– patients (**c**) (*p* = 0.68), ACPA+MRI+ vs. ACPA−MRI– patients (**c**) (*p* < 0.001) and finally ACPA+MRI− vs. ACPA+MRI+ (**c**) (*p* = 0.016). ACPA+ rheumatoid factor (RF)− patients vs. ACPA−RF− patients (**d**) (*p* = 0.30) and ACPA+RF+ patients vs. ACPA−RF− patients (**d**) (*p* = 0.006)

First a comparison was made between all ACPA-positive patients and ACPA-negative patients: ACPA-positive patients had higher erosion scores than ACPA-negative patients (*p* = 0.006; Fig. 1a). Also the presence of MRI-detected subclinical inflammation was associated with higher erosion scores (*p* < 0.001; Fig. 1b).

Next, stratification was applied for both ACPA and local subclinical joint inflammation. After this stratification, it was observed that in the absence of local subclinical inflammation ACPA-positive (ACPA+/MRI−) patients did not have higher erosion scores than ACPA-negative (ACPA−MRI−) patients (*p* = 0.68). In contrast, ACPA-positive patients with local inflammation (ACPA+/MRI+) did have higher erosion scores than ACPA-negative patients without local inflammation (ACPA−/MRI−; *p* < 0.001; Fig. 1c). Furthermore, comparing ACPA-positive patients without local inflammation (ACPA+/MRI−) to ACPA-positive patients with local inflammation (ACPA+/MRI+) revealed that the latter group had significantly higher erosions scores (*p* = 0.016, Fig. 1c). This suggests that ACPA with concomitant inflammation, but not ACPA “alone”, was associated with higher erosion scores.

When “any inflammation” (considering inflammation positive if either local subclinical joint inflammation was present or CRP was elevated) was studied, stratified analyses revealed similar results (Additional file 1: Figure S1). Also here, patients that had ACPA and inflammation had higher erosion scores, in contrast to patients that had ACPA without concomitant inflammation (*p* = 0.056).

ACPA levels within ACPA-positive patients (comparing tertiles) were not associated with erosion scores (Additional file 1: Figure S2).

### Mediation analyses; local inflammation is in the causal path of ACPA and erosions

**Fig. 2:**
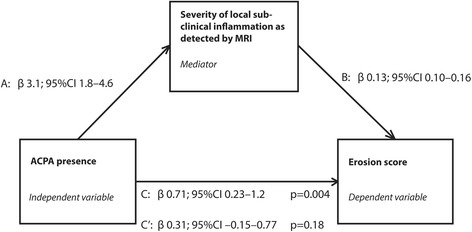
Mediation analyses showing that inflammation is in the causal pathways of anti-citrullinated protein antibodies (ACPA). Schematic overview of the causal paths that were studied using mediation models as described by Baron and Kenny. The diagram illustrates the two causal paths that can lead to the outcome; a direct path from the independent to the outcome (C) and an indirect path from the mediator to the outcome (B). Finally, there is a path between the independent variable and the mediator (A). According to the description of Baron and Kenny, to test for mediation the following three regression analyses need to be performed [16]: (1) regress the mediator on the independent variable (A) - the independent variable should significantly affect the mediator; (2) regress the dependent (outcome) variable on the independent variable (C) - also here the independent variable should significantly affect the outcome; (3) regress the dependent variable on both the mediator and the independent variable (B and C′ in one model); in the case of mediation the mediator is significantly associated with the outcome and the effect of the independent variable on the outcome is less than in step 2 (partial mediation) or there is no effect at all (full mediation). In this study, the hypothesis was tested whether severity of local inflammation detected with magnetic resonance imaging (MRI) acts as a mediator in the causal path of the presence of anti-citrullinated protein antibodies (ACPA) on the erosion score. The data revealed that inflammation mediated the effect of ACPA on bone erosions. The mediator could account for more than half of the total effect: (A*B)/(A*B + C′) = 0.57

We studied whether local inflammation is intermediary in the causal path of ACPA and erosions in three steps using mediation analyses. In linear regression analysis (Fig. 2), the presence of ACPA was significantly associated with erosion score (β 0.72; 95% CI 0.23–1.2; *p* = 0.004). Likewise, presence of ACPA was also significantly associated with the severity of local inflammation (β 3.2; 95% CI 1.8–4.6; *p* < 0.001). Importantly, presence of ACPA no longer had a significant effect on the erosion score when corrected for inflammation (β 0.31; 95% CI − 0.15 to 0.77; *p* = 0.18). Together, these results indicate that subclinical inflammation is a mediator acting in the causal path of ACPA leading to erosions and the mediator could account for more than half of the total effect: (A*B)/(A*B + C′) = 0.57.

### ACPA in the presence of RF, but not ACPA alone, associated with higher erosion scores

ACPA-positive patients with local inflammation were more often RF-positive (81%) than ACPA-positive patients without local inflammation (67%, *p* = 0.28). Stratification was therefore applied for ACPA and RF: studying the combinations of ACPA positivity and RF positivity revealed that ACPA-positive/RF-negative patients had similar erosion scores as did ACPA-negative/RF-negative patients (*p* = 0.30). However, patients having both ACPA and RF had significantly higher erosion scores (*p* = 0.006; Fig. 1d) as compared to ACPA-negative/RF-negative patients.

**Fig. 3:**
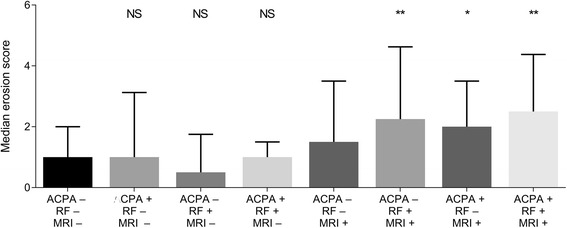
Median erosion scores in patients with Clinically Suspect Arthralgia with triple stratification for anti-citrullinated protein antibodies (ACPA), rheumatoid factor (RF) and local joint inflammation. Median erosion scores are shown with the upper limit of the interquartile range (75th percentile): *p < 0.01 compared to the ACPA−RF− magnetic resonance imaging (MRI)−group; **p < 0.01 as compared to the ACPA–RF–MRI– group; NS, non-significant as compared to the ACPA–RF–MRI– group. The following comparisons were made: ACPA−RF−MRI− patients (median erosion score 1.0) vs. ACPA+RF−MRI− (median 1.0; *p* = 0.85), ACPA−RF−MRI− vs. ACPA−RF + MRI− (median 0.5; *p* = 0.35) and ACPA−RF−MRI− vs. ACPA+RF + MRI− (median 1.0; *p* = 0.65). ACPA+RF + MRI− patients (median 1.0) vs ACPA−RF + MRI− patients (median 0.5; *p* = 0.91). Next, ACPA−RF−MRI− patients were compared to ACPA+RF−MRI+ (median 2.0; *p* = 0.033) and ACPA−RF + MRI+ patients (median 2.25; *p* = 0.001). Finally, ACPA+RF + MRI+ patients were compared to ACPA−RF−MRI− patients (median 2.5 versus 1.0; *p* < 0.0001) and ACPA+RF + MRI− patients (median 2.5 versus 1.0; *p* = 0.039). The number of patients in each group was as follows: ACPA−RF−MRI− (*n* = 214), ACPA+RF−MRI− (*n* = 4), ACPA−RF + MRI− (*n* = 26), ACPA−RF−MRI+ (*n* = 174), ACPA+RF + MRI−(*n* = 8), ACPA−RF + MRI+ (*n* = 24), ACPA+RF−MRI+ (*n* = 11) and ACPA+RF + MRI+ (*n* = 46)

Finally, triple stratification for ACPA, RF and local subclinical joint inflammation was performed (Fig. 3). First, we investigated if the single presence of ACPA or RF was associated with higher erosion scores. As compared to the ACPA−RF−MRI− reference group (median erosion score 1.0), there were no differences in patients only positive for ACPA (ACPA+RF−MRI−; median 1.0; *p* = 0.85), nor in patients single-positive for RF (ACPA−RF + MRI−; median 0.5; *p* = 0.35) or patients positive for both ACPA and RF, but without subclinical joint inflammation (ACPA+RF + MRI−; median 1.0; *p* = 0.65). ACPA+RF + MRI− patients (median 1.0) did not have significantly higher erosion scores than ACPA−RF + MRI− patients (median 0.5; *p* = 0.91). We then investigated if erosion scores were significantly higher if concomitant inflammation was present in addition to the presence of ACPA and/or RF. Compared to ACPA−RF−MRI− patients, significantly higher erosion scores were observed in ACPA-positive patients with concurrent inflammation (ACPA+RF−MRI+; median 2.0; *p* = 0.033), and in RF-positive patients with concomitant inflammation (ACPA−RF + MRI+; median 2.25; *p* = 0.001). Finally, we studied the erosion scores in ACPA+RF+ patients. Whereas ACPA+RF + MRI− patients did not have higher erosion scores than the reference group, ACPA+RF + MRI+ patients did have higher erosion scores than the ACPA−RF−MRI− patients (median 2.5 versus 1.0; *p* < 0.0001). The erosion score of the ACPA+RF + MRI+ patients was also higher than that of the ACPA+RF + MRI− patients (median 2.5 versus 1.0; *p* = 0.039). Together these data showed that the presence of ACPA and/or RF is only associated with higher erosion scores if concomitant inflammation is present.

## Discussion

This study evaluated associations between ACPA, RF, (local) subclinical joint inflammation and erosions in patients with arthralgia at risk of RA. Presence of ACPA alone, without inflammation, was not associated with higher erosion scores, in contrast to the combined presence of ACPA and inflammation. Mediation analyses revealed that local inflammation was intermediary in the causal path to erosions. These results indicate that joint inflammation has a role in the development of erosions in ACPA-positive individuals, and suggest that findings in vitro or mouse models on the independent effect of ACPAs on erosions are in contrast to findings in humans.

Furthermore, the combination of ACPA and RF, rather than presence of ACPA alone, was associated with erosions in patients with arthralgia. These results align with those obtained in patients with early rheumatoid arthritis [8, 9] and fuel the hypothesis that ACPAs alone are not the main and/or single pathogenic factor contributing to joint erosions. Although one can speculate how - or if - ACPAs contribute to joint erosions together with inflammation, results from association studies do not allow conclusions on biological mechanisms.

Our results suggest that in addition to ACPA, local joint inflammation is required for more severe erosive disease. Based on the mediation analysis we cannot definitely differentiate between full or partial mediation; the significance for ACPA from step 1 was lost in step 3 suggesting full mediation. However, as the beta was not zero, partial mediation cannot be excluded. Nonetheless, results of the mediation analyses supported the notion that erosions in ACPA-positive arthralgia rarely occurred without concomitant inflammation. This finding is in line with a previous study that showed that increased levels of CD19+ B cells and CXCL13 were observed in ACPA-positive RA and were associated with erosive disease [18].

In this study, the use of sensitive high-quality MRI data allowed us to detect erosions in a population in which the total burden of erosions is relatively low. In contrast to the setting of early inflammatory arthritis where all patients have current or recent joint inflammation, the arthralgia setting allows comparison of patients with and without inflammation.

Not all patients considered at risk of RA will develop arthritis over time, even though ACPA or (subclinical) inflammation might be present. However, because we addressed whether ACPA can directly mediate bone loss with/without concurrent inflammation, the study could be performed independent of the final clinical diagnosis.

The subgroups obtained after stratification were small in some cases (especially the ACPA+RF−MRI− subgroup after triple stratification), which could lead to underpowered analyses and the possibility of not identifying statistically significant differences. However, all analyses showed that erosion scores are highest when both ACPA and inflammation are present simultaneously, which strengthens the overall findings. Finally, our study cannot address the question as to whether the results are different for specific ACPA reactivity, as the presence of ACPA was evaluated using the commercially available CCP2 test.

We studied erosions in humans because a direct effect of ACPA on erosions has been suggested [5]. Although loss of trabecular bone as observed in mice may be dissimilar from periarticular-located erosions in humans, including the underlying mechanisms, our results indicate that ACPAs do not directly contribute to the formation of bone erosions, one of the hallmarks of RA.

## Conclusions

In conclusion, the present data in patients with arthralgia showed that erosions are associated with the combined presence of ACPA and RF, rather than with ACPA alone, and preferentially occur in patients with joint inflammation.

## Additional file

Additional file 1: **Methods.** MRI scanning and scoring. **Figure S1.** Median erosion scores in patients with Clinically Suspect Arthralgia comparing ACPA-positive and ACPA-negative patients in relation to the concomitant presence of any inflammation. **Figure S2.** Median erosion scores of ACPA-positive patients with Clinically Suspect Arthralgia according to tertiles of ACPA levels. (DOCX 133 kb)

## Abbreviations

ACPA: Anti-citrullinated protein antibodies
BME: Bone marrow edema
CI: Confidence interval
CSA: Clinically Suspect Arthralgia
ICC: Interclass correlation coefficient
IQR: Interquartile range
MCP: Metacarpophalangeal
MRI: Magnetic resonance imaging
MTP: Metatarsophalangeal
RA: Rheumatoid arthritis
RF: Rheumatoid factor
SPSS: Statistical Package for the Social Sciences
TJC: Tender joint count
