# Supplementary material for: Inflammation functions as a key mediator in the link between ACPA and erosion development: an association study in Clinically Suspect Arthralgia
Source: Arthritis Res Ther. 2018 May 3;20:89. doi: 10.1186/s13075-018-1574-3 (PMC5932781; doi:10.1186/s13075-018-1574-3)
Supplement: Supplementary file 1 — Methods. MRI scanning and scoring. Figure S1. Median erosion scores in patients with Clinically Suspect Arthralgia comparing ACPA-positive and ACPA-negative patients in relation to the concomitant presence of any inflammation. Figure S2. Median erosion scores of ACPA-positive patients with Clinically Suspect Arthralgia according to tertiles of ACPA levels. (DOCX 133 kb) [file 13075_2018_1574_MOESM1_ESM.docx]

# Additional file 1: Methods. MRI scanning and scoring

## Detailed MR-scan protocol

MR imaging was performed on an MSK-extreme 1.5T extremity MR imaging system (GE, Wisconsin, USA) using a 145mm coil for the foot and a 100mm coil for the hand. The patient was positioned in a chair beside the scanner, with the hand or foot fixed in the coil with cushions.

In the hand, the following sequences were acquired before contrast injection: T1-weighted fast spin-echo (FSE) sequence in the coronal plane (repetition time (TR) 575 ms, echo time (TE) 11.2 ms, acquisition matrix 388×288, echo train length (ETL) 2). After intravenous injection of gadolinium contrast (gadoteric acid, Guerbet, Paris, France, standard dose of 0.1 mmol/kg) the following sequences were obtained: T1-weighted FSE sequence with frequency selective fat saturation (fatsat) in the coronal plane (TR/TE 700/9.7ms, acquisition matrix 364×224, ETL 2), T1-weighted FSE sequence with frequency selective fat saturation in the axial plane (wrist: TR/TE 540/7.7 ms; acquisition matrix 320x192; ETL 2 and metacarpophalangeal joints: TR/TE 570/7.7 ms; acquisition matrix 320x192; ETL 2).

The obtained sequences of the forefoot were post-gadolinium sequences which included: T1-weighted FSE fatsat sequence in the axial plane (TR/TE 700/9.5ms; acquisition matrix 364x224, ETL 2) and: T1-weighted FSE fatsat sequence in the coronal plane (perpendicular to the axis of the metatarsals) (TR/TE 540/7.5ms; acquisition matrix 320x192, ETL 2).

Field-of-view was 100mm for the hand and 140mm for the foot. Coronal sequences of the hand had 18 slices with a slice thickness of 2mm and a slice gap of 0.2mm. Coronal sequences of the foot had 20 slices with a slice thickness of 3mm and a slice gap of 0.3mm. All axial sequences had a slice thickness of 3mm and a slice gap of 0.3mm with 20 slices for the wrist, 16 for the metacarpophalangeal joints and 14 for the foot.

According to the RAMRIS-method, T2-weighted fat suppressed sequences, or when this sequence is not available a short tau inversion recovery (STIR) sequence, should be used to assess Bone Marrow (O)Edema (BME). Previously, three studies have demonstrated that a contrast enhanced T1-weigthed fat suppressed sequence has a strong correlation with T2-weighted fat suppressed sequences.^1–3^ A T2-weighted image shows increased water signal and a contrast-enhanced T1-weighted sequence shows increased water content and the increased perfusion and interstitial leakage. A strong correlation has been shown in arthritis patients but also in patients without inflammatory diseases such as bone bruises, intraosseous ganglions, bone infarcts and even nonspecific cases.^2,3^ We used the contrast enhanced T1-weighted fat suppressed sequence as it allowed a shorter scan time and has a higher signal to noise ratio.

In the first 78 patients a T1-weighted sequence and a T2-weighted fat saturated sequence were acquired in the axial plane (relative to the anatomical position), before contrast agent administration. In the remaining patients postcontrast, T1-weighted, fat saturated sequences were acquired in axial and coronal planes. This provided more information while reducing scanning-times.

## MR scoring

All bones, joints and tendons were scored semi-quantitatively. Similar to the RAMRIS method, synovitis score was scored based on the volume of enhancing tissue in the synovial compartment (none, mild, moderate, severe (range 0-3)). Erosions were scored, according to RAMRIS, based on the affected volume of the bone on a score from 0-10 (no erosions, 0-10%, 10-20%, etc.). Similar to method described by Haavardsholm et al the tenosynovitis-score was based on the thickness of peritendinous effusion or synovial proliferation with contrast enhancement (normal, <2mm, 2-5mm, >5mm (range 0-3)).^4,5^ BME was depicted on a contrast enhanced T1-weigthed fat suppressed sequence and also scored on a 0-3 scale based on the affected volume of the bone (no BME, >0-33%, >33-66%, >66%), The scores of all joints were summed and the total BME, synovitis and tenosynovitis scored were summed as well, yielding the total MRI-inflammation-score. As described previously^6^, an MRI was considered positive if each of two readers indicated inflammation in at least 1 joint (synovitis, BME or tenosynovitis) that was present in <5% of the healthy persons in the same age-category at the same location, called the ‘5% corrected definition’.^7^ For example, a 65-year old patient with grade 1 synovitis in MCP-4 was indicated positive for the ‘uncorrected definition’, positive for the ‘5% corrected definition’ as it was seen in 4% of controls in this age category on this location and negative for the ‘1% corrected definition’. Likewise, a 65-year old patient with grade 1 tenosynovitis of the flexor of MCP-3 was negative for the ‘uncorrected definition’ and for the ‘5% and 1% corrected definitions’ as it was seen in 12% of controls.^7^

## **References**

1. Stomp W, Krabben A, Heijde D van der, *et al.* Aiming for a shorter rheumatoid arthritis MRI protocol: can contrast-enhanced MRI replace T2 for the detection of bone marrow oedema? *Eur Radiol* 2014;24:2614–22.

2. Schmid MR, Hodler J, Vienne P, *et al.* Bone Marrow Abnormalities of Foot and Ankle: STIR versus T1-weighted Contrast-enhanced Fat-suppressed Spin-Echo MR Imaging. *Radiology* 2002;224:463–9.

3. Mayerhoefer ME, Breitenseher MJ, Kramer J, *et al.* STIR vs. T1-weighted fat-suppressed gadolinium-enhanced MRI of bone marrow edema of the knee: Computer-assisted quantitative comparison and influence of injected contrast media volume and acquisition parameters. *J Magn Reson Imaging* 2005;22:788–93.

4. Østergaard M, Edmonds J, McQueen F, *et al.* An introduction to the EULAR–OMERACT rheumatoid arthritis MRI reference image atlas. *Ann Rheum Dis* 2005;64:i3–7.

5. Haavardsholm EA, Østergaard M, Ejbjerg BJ, *et al.* Introduction of a novel magnetic resonance imaging tenosynovitis score for rheumatoid arthritis: reliability in a multireader longitudinal study. *Ann Rheum Dis* 2007;66:1216–20.

6. Van Steenbergen HW, Mangnus L, Reijnierse M, *et al*. Clinical factors, anticitrullinated peptide antibodies and MRI-detected subclinical inflammation in relation to progression from clinically suspect arthralgia to arthritis. *Ann Rheum Dis*. 2016 Oct;75(10):1824-30.

7. Mangnus L, Van Steenbergen HW, Reijnierse M, van der Helm-van Mil AHM. MR-detected features of inflammation and erosions occur in symptom-free persons from the general population. *Arthritis Rheumatol*. 2016 Nov;68(11):2593-2602.

# Additional file 1: Figure S1. Histograms showing median erosion scores of patients with Clinically Suspect Arthralgia comparing ACPA-positive and ACPA-negative patients in relation to the concomitant presence of any inflammation.


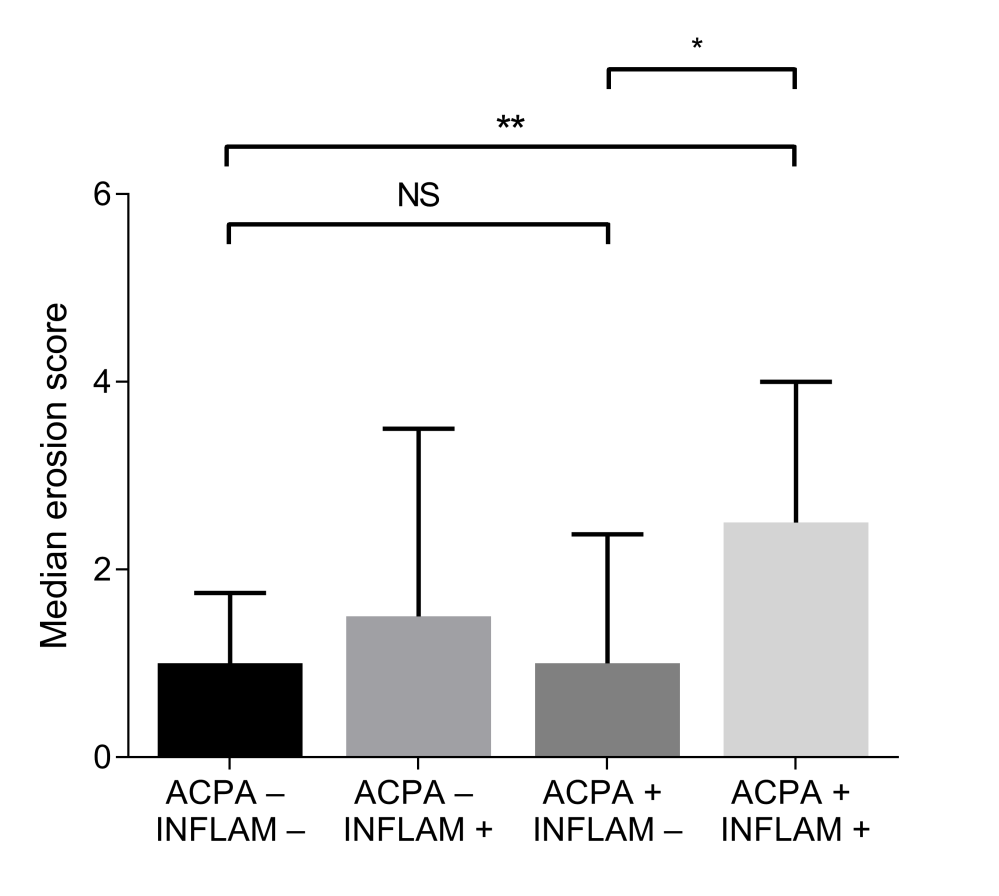


**Legend:**

Histograms showing median erosion scores with the upper limit of the interquartile range (75^th^ percentile). Any inflammation was defined as either the presence of local subclinical inflammation in MCP, wrist or MTP joints and/or the presence of elevated C-Reactive Protein. ** indicates significance of p<0.01 level, * indicates significance of p<0.05 level, NS indicates non-significance. The following comparisons have been made: ACPA–INFLAM– vs. ACPA+INFLAM– (p=0.82), ACPA–INFLAM–vs. ACPA+INFLAM+ (p<0.001) and finally ACPA+INFLAM– vs. ACPA+MRI+ patients (p=0.41).

# Additional file 1: Figure S2. Histograms showing median erosion scores of ACPA-positive Clinically Suspect Arthralgia patients according to tertiles of ACPA levels.


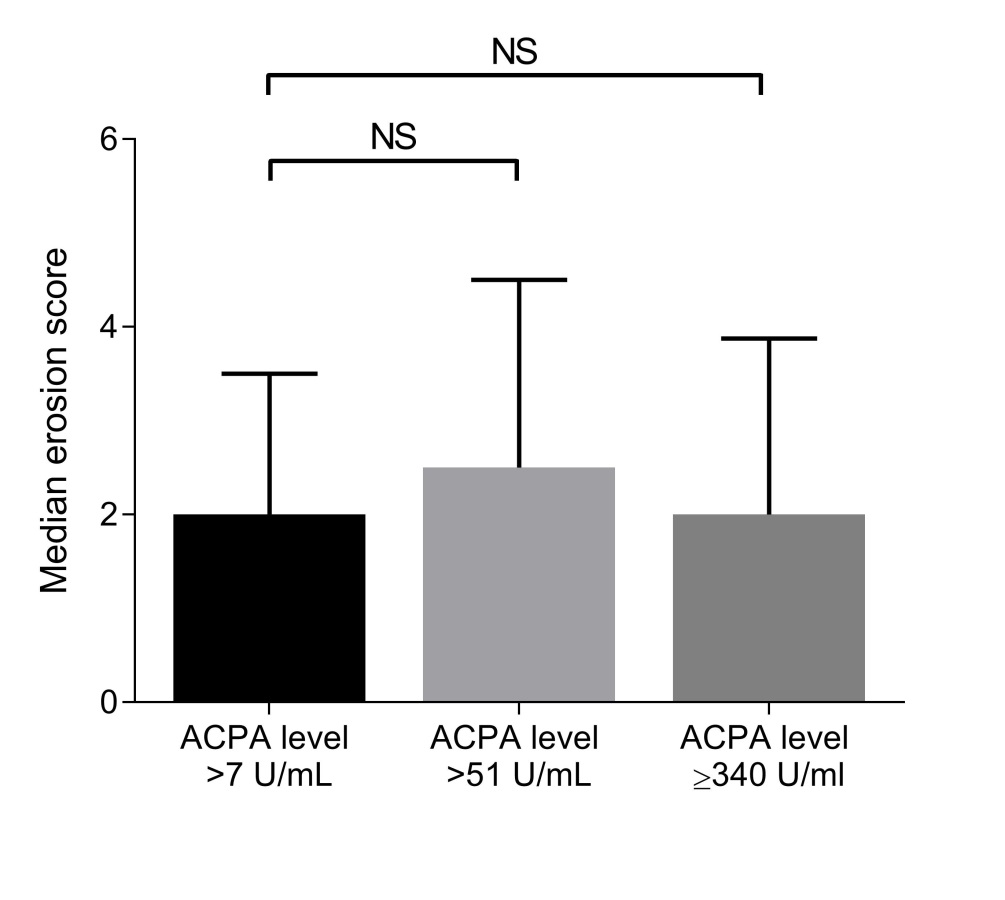


**Legend:**

Histograms showing median erosion scores with the upper limit of the interquartile range (75^th^ percentile). Kruskal-Wallis test for overall difference between groups showed no significance (p=0.99). No stratification was applied for the absence/presence of local joint inflammation in the presented figure. NS indicates non-significance.

When stratification was applied for local joint inflammation, similarly no differences were found between the different levels of ACPA. When local subclinical inflammation was absent: ACPA levels of >7-≤51 vs >51-<340 U/ml: p=0.86 and ACPA levels of >51-<340 vs ≥340 U/ml: p=0.65. When local subclinical inflammation was absent: ACPA levels of >7-≤51 vs >51-<340 U/ml: p=0.53 and ACPA levels of >51-<340 vs ≥340 U/ml: p=0.98.
